# Associations of Sperm mtDNA Copy Number, DNA Fragmentation Index, and Reactive Oxygen Species With Clinical Outcomes in ART Treatments

**DOI:** 10.3389/fendo.2022.849534

**Published:** 2022-03-23

**Authors:** Wei-Hui Shi, Mu-Jin Ye, Ning-Xin Qin, Zhi-Yang Zhou, Xuan-You Zhou, Nai-Xin Xu, Song-Chang Chen, Shu-Yuan Li, Chen-Ming Xu

**Affiliations:** ^1^ International Peace Maternity and Child Health Hospital, School of Medicine, Shanghai Jiao Tong University, Shanghai, China; ^2^ Shanghai Key Laboratory of Embryo Original Diseases, Shanghai, China; ^3^ Department of Assisted Reproductive Medicine, Shanghai First Maternity and Infant Hospital, School of Medicine, Tongji University, Shanghai, China; ^4^ Obstetrics and Gynecology Hospital, Institute of Reproduction and Development, Fudan University, Shanghai, China

**Keywords:** sperm mitochondrial DNA copy number, DNA fragmentation index, reactive oxygen species, sperm quality, assisted reproductive technology

## Abstract

Recent studies have suggested that sperm mitochondrial DNA copy number (mtDNA-CN), DNA fragmentation index (DFI), and reactive oxygen species (ROS) content are crucial to sperm function. However, the associations between these measurements and embryo development and pregnancy outcomes in assisted reproductive technology (ART) remain unclear. Semen samples were collected from 401 participants, and seminal quality, parameters of sperm concentration, motility, and morphology were analyzed by a computer-assisted sperm analysis system. DFI, mtDNA-CN, and ROS levels were measured using sperm chromatin structure assay, real-time quantitative polymerase chain reaction, and ROS assay, respectively. Among the participants, 126 couples underwent ART treatments, including *in vitro* fertilization (IVF) and intracytoplasmic sperm injection (ICSI), and 79 of the couples had embryos transferred. In 401 semen samples, elevated mtDNA-CN and DFI were associated with poor seminal quality. In 126 ART couples, only mtDNA-CN was negatively correlated with the fertilization rate, but this correlation was not significant after adjusting for male age, female age, seminal quality, ART strategy, number of retrieved oocytes, controlled stimulation protocols, and cycle rank. Regarding pregnancy outcomes, sperm mtDNA-CN, ROS, and DFI were not associated with the clinical pregnancy rate or live birth rate in 79 transferred cases. In conclusion, increased mtDNA-CN and DFI in sperm jointly contributed to poor seminal quality, but sperm mtDNA-CN, ROS, and DFI were not associated with clinical outcomes in ART.

## Introduction

It is estimated that over 186 million people are affected by infertility, and assisted reproductive technology (ART) treatments are continuously increasing worldwide (1). However, there is a challenge in that a large proportion of embryos during ART end with adverse outcomes, such as implantation failures and miscarriages (2). Abnormal gametes from parents impact embryo quality, and the effect of paternal factors has been increasingly explored in recent years (3, 4).

Previous studies have investigated the associations between seminal quality, including parameters of sperm concentration, motility, and morphology, and ART outcomes. It has been reported that both sperm concentration and motility are strongly correlated with the fertilization rate, and poor seminal quality may lead to developmental failures of embryos, mainly manifested as blastomere fragmentations (5). However, sperms with normal morphology were selected for the intracytoplasmic sperm injection (ICSI) process, and sperm concentration rarely affects this treatment. Thus, sperm motility plays a more significant role in embryo development in ICSI (6).

Researchers have investigated the relationship between sperm structural abnormalities and adverse pregnancy outcomes (7–9). As an indicator of sperm chromatin integrity, the DNA fragmentation index (DFI) has been suggested to have a negative correlation with embryo development and implantation rates in ICSI cycles (9), while some studies have indicated contrary results (10). Sperm mitochondria are essential to the normal reproductive process, as they are involved in multiple functions, including the production of adenine triphosphate and reactive oxygen species (ROS) as well as the regulation of apoptosis (11). Oxidative stress induced by excess ROS in sperm has been found to impair DNA demethylation in the paternal pronucleus and affects embryo development (8). A negative association has been observed between seminal ROS and pregnancy rates after *in vitro* fertilization (IVF) (12). Additionally, sperm mitochondrial DNA copy number (mtDNA-CN), a relative measure of mtDNA content, has also been reported to be negatively correlated with the fertilization rate (13). Rosati et al. (14) found that higher sperm mtDNA-CN is associated with lower pregnancy probability in couples without contraception, suggesting that mtDNA might be a potential clinical biomarker to predict male fecundity.

It remains unclear whether the measurements mentioned above, namely, DFI, mtDNA-CN, and ROS, are predictive of ART outcomes. Therefore, the present study aimed to investigate the correlations of sperm mtDNA-CN, ROS, and DFI with male fertility, embryo viability, and pregnancy outcomes.

## Methods

### Subjects

All participants were recruited from July 2020 to September 2020 from the Outpatient Department of the Reproductive Center at the International Peace Maternity and Child Health Hospital (IPMCH), Shanghai Jiao Tong University School of Medicine. The inclusion criteria were as follows: 1) 20–50 years of age; 2) no *AZF* gene microdeletions; 3) no mycoplasma or chlamydia infections; 4) no medication taken within 3 months; and 5) sperm concentration ≥1 million/ml. Semen samples were collected by masturbation after 3–7 days of sexual abstinence. The present study was approved by the IPMCH Ethics Review Committee and performed according to the Declaration of Helsinki. Written consent forms were obtained from all participants.

### Semen Analysis and Sperm Chromatin Structure Assay

After liquefaction, sperm concentration and motility were assessed by the computer-assisted sperm analysis (CASA) system (Hamilton Thorne IVOS II, USA), and sperm morphology was evaluated with Papanicolaou staining according to the World Health Organization (WHO) laboratory manual (15). Sperm DFI was determined by a sperm chromatin structure assay (8). In detail, sperm samples were first diluted to a concentration of 2 × 10^6^/ml with TNE buffer (0.01 M Tris–HCl, pH 7.4, 0.15 M NaCl, 1 mM EDTA). Then, 100 µl of diluted sperm suspension was mixed with 200 µl of acid-detergent solution (pH 1.2; 0.08 N HCl, 0.15 M NaCl, 0.1% Triton X-100) and incubated for 30 s on ice. After adding 600 µl of acridine orange, the sample was incubated for 3 min and analyzed by the NovoCyte Flow Cytometer (Agilent, CA, USA).

### Sperm DNA Extraction

Semen samples were washed three times with 1× PBS and centrifuged at 200 g for 5 min. Somatic cells were eliminated with 0.1% sodium dodecyl sulfate (3250GR500, BioFroxx) and 0.5% Triton™-X100 (X100, Sigma) in diethypyrocarbonate (DEPC)-treated water (AM9920, Invitrogen) at 4°C for 15 min. The spermatozoa were then homogenized with 1-mm beads in Tissue Lysis buffer (69504, Qiagen) containing 10 mg/ml Proteinase K and 150 mM DL-dithiothreitol (A100281, Sangon Biotech, China). Total sperm DNA was extracted using the DNeasy Blood & Tissue Kit (69504, Qiagen) following the manufacturer’s instructions.

### Quantification of mtDNA Copy Number

The mtDNA-CN was measured by real-time quantitative polymerase chain reaction (qPCR) using a QuantStudio™ 7 Flex real-time PCR machine (4485701, Applied Biosystems) (16). Briefly, TaqMan primers were designed in a stable segment in the minor arc of mtDNA (mtMinArc), and RNAse P (4403326, ThermoFisher, USA) was used as the genomic DNA reference. The detailed primer sequences are shown in **Supplementary Table S1**. Real-time PCR with three technical replicates was performed as previously described (16). The mtDNA-CN was calculated using the following formula: mtDNA-CN = 2^△CT (mtDNA-CN)^, where △CT (mtDNA-CN) = CTRNase P − CTmtMinArc.

### Determination of Sperm Reactive Oxygen Species Levels

Sperm ROS content was measured by a ROS assay kit (S0033M, Beyotime Biotech, Shanghai, China) following the manufacturer’s instructions. The collected sperm samples were washed three times with 1× PBS (200 g, 5 min) and then incubated with 2’,7’-dichlorodihydrofluorescein diacetate (DCFH-DA; 10 μmol/L) at 37°C for 20 min. The samples were then washed three times with 1× PBS (200 g, 5 min), and the fluorescent signals of DCFH-DA oxidized products [2’,7’-dichlorofluorescein (DCF)] were detected using a Synergy™ H1 Microplate Reader (BioTek Instruments, Inc., Vermont, USA) under 488-nm excitation. To normalize the ROS level, ROS per million sperm (ROS/MS) was used to represent the average ROS content in each seminal sample.

### Assisted Reproductive Technology Procedure

Different protocols of controlled ovarian stimulation (COS), including gonadotrophin-releasing hormone agonist (GnRH-a) protocols (long, short, and ultra-long protocols), the GnRH antagonist protocol, and mild ovarian stimulation, were performed in 126 couples according to the women’s age, ovarian reserve, and previous IVF outcomes (**Supplementary Table S2**). IVF of oocytes was carried out through inseminating oocytes with motile sperm or injecting a single sperm into the cytoplasm of an oocyte. The zygotes were cultured to blastocysts on Day 5 and freshly transferred into the uterus or cryopreserved and thawed at a suitable time of embryo transfer.

### Outcome Assessment

In terms of ART, only those IVF/ICSI cycles performed within 3 months after the semen analysis were included. The embryonic outcomes included the fertilization rate, cleavage rate, and top-quality embryo rate. The fertilization rate was defined as the number of two pronuclear embryos devised by the retrieved cumulus–oocyte complex. The cleavage rate was the percentage of cleaving embryos on Day 3 in all fertilized oocytes. The cleaving zygotes were classified into Grades 1–5 according to the numbers and sizes of blastomeres and the percentage of cytoplasmic fragments (17). The top-quality embryo rate was calculated as the number of embryos evaluated as Grade 1 or 2 divided by the number of total embryos. In terms of embryos transferred into the uterus, pregnancy outcomes were evaluated in every transfer cycle. Clinical pregnancy was defined as the presence of an intrauterine gestational sac by ultrasound examination at a gestational age of 7 weeks.

### Statistical Analysis

The characteristics of the participants were summarized. Continuous variables are presented as the median (interquartile range) and were compared with the Mann–Whitney U test. Categorical variables are presented as percentages and were compared using the chi-square test. Seminal quality was categorized as normal or abnormal sperm group. Sperm with at least one parameter, including sperm concentration, motility, and morphology, lower than the WHO criteria was categorized as abnormal sperm (concentration ≥15 × 10^6^ per ml; total motility ≥40%; normal forms ≥4%). The associations between the three measurements (mtDNA-CN, ROS/MS, and DFI) and seminal quality were analyzed by binary logistic regression. Embryonic outcomes were analyzed with linear regression. The generalized linear model (GLM) was performed to adjust the covariates, including male age, female age, seminal quality, number of retrieved oocytes, ART strategy, and cycle rank. Regarding pregnancy outcomes, generalized estimating equations (GEEs) were used to address the correlation of different transfer cycles in the same patient. Odds ratios (ORs) and 95% confidence intervals (CIs) were calculated for the variates in the model. All statistical analyses were conducted with SPSS statistics 24 (IBM, Chicago, USA).

## Results

### Demographic and Biochemical Characteristics of Participants

During the study period, 401 male participants who fulfilled the inclusion criteria were included in our study. Among them, 126 with their spouses were subjected to ART within 3 months after the semen analysis in our center. The demographic characteristics, sperm parameters, and three measurements are summarized in **Table 1**. Compared to the IVF cases, the ICSI cases showed lower total motility and higher DFI levels (*P* < 0.05). For controlled ovarian stimulation, 80 cases underwent the GnRH antagonist protocol, 27 cases underwent mild ovarian stimulation, and 19 cases underwent GnRH agonist protocols, including long, short, and ultra-long protocols (**Supplementary Table S2**).

**Table 1:** Sperm parameters and measurements in all participants and ART cases.

	All participants (n = 401)	IVF cases (n = 82)	ICSI cases (n = 44)	P*
Male age, years	33 (31, 37)	33 (30, 36)	35.5(32, 40)	0.009
Female age, years		33 (29, 36)	34 (31, 38)	0.080
Percentage of male with abnormal sperms	71%	55%	59%	0.708
Morphology, %	3 (2, 4)	3 (2, 4)	3 (1, 4)	0.244
Sperm concentration, million/ml	36.99 (21.48, 59.29)	40.62 (26.21, 60.11)	36.26 (18.85, 53.64)	0.250
Total motility, %	41.90 (26.85, 52.30)	42.95 (29.75, 51.60)	34 (17.65, 49.05)	0.014
mtDNA-CN	3.80 (2.10, 7.38)	3.30 (1.96, 8.36)	4.37 (2.39, 9.07)	0.321
ROS/MS	81.33 (48.65, 174.80)	74.15 (49.89, 146.10)	73.00 (47.23, 154.66)	0.976
DFI	9.92 (5.61, 15.86)	7.98 (5.16, 14.56)	11.83 (6.59, 22.71)	0.005

The data were analyzed with the Mann–Whitney U test.

mtDNA-CN, mitochondrial DNA copy number; ROS/MS, reactive oxygen species per million sperm; DFI, DNA fragmentation index.

*P value indicates the comparison between IVF and ICSI cases.

ART, assisted reproductive technology; IVF, in vitro fertilization; ICSI, intracytoplasmic sperm injection.

### Associations of Sperm mtDNA-CN, DNA Fragmentation Index, and Reactive Oxygen Species With Seminal Quality

**Figure 1:**
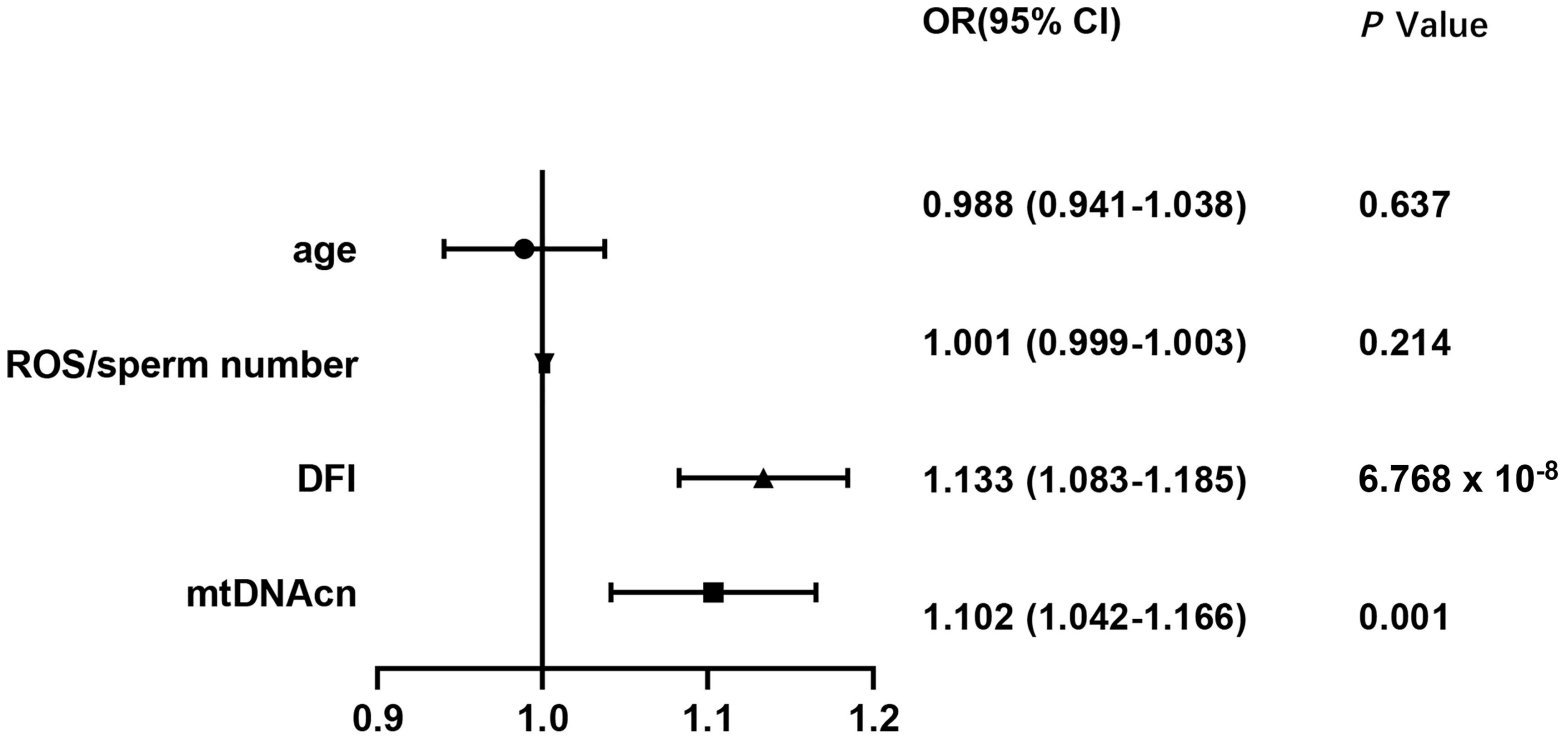
Associations of mtDNA-CN, DFI, ROS, and male age with seminal quality. mtDNA-CN, mitochondrial DNA copy number; ROS, reactive oxygen species; DFI, DNA fragmentation index; OR, odds ratio; CI, confidence interval.

Univariate logistic regression showed that mtDNA-CN, ROS/MS, and DFI were all negatively associated with seminal quality (**Supplementary Table S3**). Based on the fifth WHO laboratory manual (15), the seminal samples were divided into two groups as follows: the normal sperm group (n = 116) and the abnormal sperm group (n = 285). As shown in **Figure 1**, the multivariate logistic regression suggested that only the mtDNA-CN (OR 1.102, 95% CI 1.042–1.166; *P* = 0.001) and DFI (OR 1.133, 95% CI 1.083–1.185; *P* = 6.768 × 10^-8^) were associated with seminal quality.

### Correlations of Sperm mtDNA-CN, DNA Fragmentation Index, and Reactive Oxygen Species With Embryonic Outcomes

Regarding the three embryonic outcomes, a higher sperm mtDNA-CN level was associated with a lower fertilization rate in all ART cases (**Table 2**) (β = −0.629, 95% CI -1.175 to -0.083; *P* = 0.024). The quartile analysis showed a similar trend that the fourth quartile group of sperm mtDNA-CN had the lowest fertilization rate (**Table 3**). However, after adjustment for factors, including male age, female age, seminal quality, number of retrieved oocytes, ART strategy, COS protocols, and cycle rank, the adjusted model indicated that the association between sperm mtDNA-CN and fertilization rate was not significant (**Table 3**). There was no significant correlation between sperm mtDNA-CN and cleavage rate (β = 0.108, 95% CI -0.344 to 0.560; *P* = 0.637) or top-quality embryo rate (β = 0.345, 95% CI -0.345 to 1.034; *P* = 0.325) (**Table 2**).

**Table 2:** Embryonic outcomes of patients with ART.

	Fertilization rate (%)		Cleavage rate (%)		Top-quality embryo rate (%)	
	Coefficients (95% CI)	*P*	Coefficients (95% CI)	*P*	Coefficients (95% CI)	*P*
mtDNA-CN	-0.629 (-1.175, -0.083)	0.024	0.108 (-0.344, 0.560)	0.637	0.345 (-0.345, 1.034)	0.325
DFI	-0.128 (-0.567, 0.311)	0.564	0.127 (-0.229, 0.483)	0.482	0.377 (-0.165, 0.919)	0.171
ROS/MS	-0.012 (-0.035, 0.012)	0.325	0.005 (-0.013, 0.024)	0.566	0.009 (-0.20, 0.038)	0.532

The data were analyzed with linear regression (n = 126).

ART, assisted reproductive technology; mtDNA-CN, mitochondrial DNA copy number; ROS/MS, reactive oxygen species per million sperm; DFI, DNA fragmentation index; CI, confidence interval.

**Table 3:** Association of mtDNA-CN with fertilization rate.

mtDNA-CN	Fertilization rate			
	OR (95% CI)	*P*	Adj OR (95% CI) ^a^	*P*
Q1	Reference			
Q2	0.919 (0.818, 1.033)	0.158	0.921 (0.826, 1.028)	0.144
Q3	0.928 (0.826, 1.043)	0.211	0.968 (0.868, 1.079)	0.553
Q4	0.870 (0.773, 0.979)	0.021	0.915 (0.817, 1.025)	0.125

The data were analyzed with quartile analysis.

Q1–4, quartiles 1–4; OR, odds ratio.

a Adj OR was adjusted for male age, female age, seminal quality, number of retrieved oocytes, ART strategy (IVF/ICSI), COS protocols, and cycle rank.

CI, confidence interval.

Moreover, neither ROS/MS nor DFI was associated with three embryonic outcomes in all ART cases (**Table 2**). However, in 92 couples performed with the first ART cycle, there was a statistically significant association between the DFI and fertilization rate (**Supplementary Table S4**), which was not observed after adjustment for male age, female age, seminal quality, number of retrieved oocytes, ART strategy, and COS protocols (**Supplementary Table S5**).

### Pregnancy Outcomes

To date, 79 of 126 couples undergoing ART have undergone embryo transfer. In total, 114 transfer cycles were conducted, including 12 fresh embryo transfers and 102 frozen embryo transfers (**Supplementary Table S6**). In all transfer cycles, mtDNA-CN, DFI, and ROS/MS were not associated with clinical pregnancy rate and live birth rate (**Table 4**), and this lack of association remained after adjustment for male age, female age, seminal quality, types of embryo transfer (frozen or fresh), ART strategy, IVF cycle rank, COS protocols, and number of retrieved oocytes (**Supplementary Table S7**).

**Table 4:** Pregnancy outcomes of patients with embryo transfer.

	Clinical pregnancy		Live birth	
	OR (95% CI)	*P*	OR (95% CI)	*P*
mtDNA-CN	0.997 (0.943, 1.055)	0.927	1.014 (0.959, 1.071)	0.635
DFI	1.013 (0.969, 1.059)	0.575	1.011 (0.966, 1.058)	0.651
ROS/MS	1.001 (0.999, 1.004)	0.241	1.000 (0.998, 1.003)	0.784

The data were analyzed with generalized estimating equations.

mtDNA-CN, mitochondrial DNA copy number; ROS/MS, reactive oxygen species per million sperm; DFI, DNA fragmentation index; CI, confidence interval; OR, odds ratio.

## Discussion

In the present study, we found that three sperm measurements, namely, mtDNA-CN, DFI, and ROS, were negatively associated with seminal quality. In 92 cases conducted with the first ART cycle, high levels of mtDNA-CN and DFI were correlated with a poor fertilization rate. However, this correlation was not significant after adjusting for male age, female age, seminal quality, ART strategy, number of retrieved oocytes, COS protocols, and cycle rank. Moreover, pregnancy outcomes were summarized in 79 cases of transferred embryos, and no associations were found between the three measurements and the clinical pregnancy rate or the live birth rate.

During spermatogenesis, most of the cytoplasm is discarded, while a proportion of mitochondria are retained in the midpiece of mature sperm to provide energy for flagellar beating (18). The role of mitochondria in sperm function, especially sperm motility, has been widely noted. It has been reported that abnormal mitochondrial structures and functions, such as short midpieces, abnormal assemblies, and membranous defects, are associated with poor seminal quality (19, 20). As an important component of mitochondria, mtDNA encodes 22 tRNAs, two rRNAs, and 13 proteins that are crucial for oxidative phosphorylation (21). Mutations and deletions of mtDNA have been reported in asthenozoospermia and shown to be correlated with male infertility when they present beyond a certain threshold level (22). In addition, amplification of sperm mtDNA-CN has been observed in sperm samples from infertile males (23).

Consistent with previous studies in various species (23–26), higher mtDNA-CN in abnormal sperm was observed in the present study. Although the underlying mechanism remains unclear, the elevated mtDNA-CN in abnormal sperm might be explained by abnormal gene expression, mtDNA mutations/deletions, and mitochondria *per se*. First, abnormal expression of genes that regulate mtDNA transcription and replication, such as mitochondrial transcription factor A (*TFAM*), may be responsible for the elevated mtDNA-CN. Because *TFAM* expression is positively associated with mtDNA-CN and negatively correlated with sperm motility (27), increased *TFAM* expression may lead to the aberrant replication of mtDNA in abnormal sperm. Second, increased mtDNA-CN may compensate for mitochondrial dysfunction caused by mtDNA mutations or deletions (28–30). Moreover, mtDNA-CN may accumulate due to the imbalance between mitochondrial fusion and fission. In mitochondrial fission factor (*Mff*) mutant mice, sperm mitochondria fail to divide and mitochondrial sheaths are disjointed, resulting in abnormal sperm morphology and motility, which ultimately cause reduced fertility in mice (31).

ROS, a variety of oxygen-derived free radicals, is essential for sperm maturation, capacitation, hyperactivation, and acrosome reactions at low levels (32). However, excess ROS leads to oxidative stress and DNA damage in sperm, including DNA fragmentation, mtDNA damage, telomere attrition, epigenetic abnormalities, and Y chromosome microdeletions (33). In the present study, ROS/MS was correlated with mtDNA-CN in both normal and abnormal sperm groups, while the association of ROS/MS and DFI was not observed (**Supplementary Figure S1**), indicating that the negative effects of ROS on mtDNA may be more serious than those on nuclear DNA. Compared to nuclear DNA, mtDNA is adjacent to the ROS source and more susceptible to oxidative stress due to a lack of protective histones and repair system. In the multivariate logistic regression analysis, the association with ROS/MS was not significant, yet mtDNA and DFI were still associated with seminal quality, suggesting that mtDNA and DFI are more predictive of seminal quality.

For embryo and pregnancy outcomes, it has been reported that abnormal seminal quality has negative paternal effects in IVF or ICSI procedures (5, 6, 34, 35). However, the effects of sperm mtDNA-CN, DFI, and ROS on embryo quality are ambiguous. In 2019, Wu et al. (13) found that sperm mtDNA-CN, as well as mtDNA deletions, is inversely associated with the odds of fertilization and high-quality embryos after adjusting for male age and measurement batches. In addition, a prospective study of couples discontinuing contraception has revealed that a higher mtDNA-CN is associated with a lower pregnancy probability (14). In contrast, it has been recently reported that sperm mtDNA-CN is not a prognostic factor for fertilization, usable blastocyst development, or live birth rates in couples who undergo ICSI (36). In this study, mtDNA-CN is negatively associated with the fertilization rate, but the association is not significant after adjusting for male age, female age, seminal quality, number of retrieved oocytes, ART strategy, and cycle rank. Thus, further studies on the role of mtDNA-CN in embryo development are needed.

The role of DFI has been investigated over a longer time span, and many meta-analyses and systematic reviews have summarized the effect of sperm DNA damage on clinical outcomes after IVF or ICSI (37–41). Overall, these studies have suggested that there is a difference between the outcomes of IVF and ICSI. Most studies have reported no significant association of DFI and clinical outcomes in ICSI. Nevertheless, increased DFI leads to a negative impact on IVF outcomes, including fertilization rate, embryo quality, implantation rate, pregnancy rate, and live birth rate (41, 42). However, the correlation analysis of DFI and IVF outcomes in these studies was not adjusted for factors, such as seminal quality, COS protocols, and number of retrieved oocytes. In the present study, the correlation of DFI with fertilization rate was also observed in the first ART cycle of couples, but this correlation was not statistically significant in the adjusted model. Notably, in the study by Pregl Breznik et al. (42), washed sperm samples during IVF procedures were analyzed for hyaluronan-binding assays, DFI, and hyperactivity, which was a good strategy to attenuate the impact of fluctuations in sperm measurements on clinical outcomes.

Sperm ROS was reported to have a greater effect on embryo development than the fertilization process (43). However, negative associations of ROS with fertilization rate and pregnancy rate have also been reported (12, 44). In the present study, however, ROS/MS was not correlated with the fertilization rate, cleavage rate, top-quality embryo rate, or clinical pregnancy rate.

The present study prospectively investigated the relationships between three sperm measurements and clinical outcomes throughout the ART procedure, including seminal quality, fertilization rate, cleavage rate, clinical pregnancy rate, and live birth rate. A major limitation of this study was the limited sample size for the analysis of embryonic/pregnancy outcomes. Moreover, the sperm samples analyzed for the three measurements and sperm quality were collected before the ART procedure. Even though the analysis of IVF/ICSI cycles was restricted to within 3 months after the semen analysis, the fluctuations in sperm measurements and parameters could not be ignored. Multicenter studies with a larger sample size are warranted to validate these findings in the future.

## Conclusions

In conclusion, sperm mtDNA-CN, ROS/MS, and DFI were separately associated with sperm parameters, while elevated sperm mtDNA-CN and DFI jointly contributed to poor seminal quality. Moreover, mtDNA-CN was negatively correlated with fertilization rate in ART cases, which was not significant after adjusting for male age, female age, seminal quality, ART strategy, number of retrieved oocytes, COS protocols, and cycle rank. For pregnancy outcomes, sperm mtDNA-CN, ROS/MS, and DFI were not associated with clinical pregnancy rate or live birth rate. Further studies are necessary to determine the role of sperm mtDNA-CN, ROS/MS, and DFI in embryonic and fetal development.

## Data Availability Statement

The original contributions presented in the study are included in the article/**Supplementary Material**. Further inquiries can be directed to the corresponding authors.

## Ethics Statement

The studies involving human participants were reviewed and approved by the International Peace Maternity and Child Health Hospital Ethics Review Committee. The patients/participants provided their written informed consent to participate in this study.

## Author Contributions

WHS and MJY collected seminal samples and wrote the article. ZYZ and NXQ performed the experiments and analyzed the data. XYZ, NXX, and SCC collected the clinical outcomes of the participants. SYL and CMX designed the study and revised the article. All authors contributed to the article and approved the submitted version.

## Funding

This work was supported by the National Natural Science Foundation of China (Nos. 81771638, 81971344, 81871136, and 81501231) and the Shanghai Municipal Health Commission (No. GW-10.1-XK07).

## Conflict of Interest

The authors declare that the research was conducted in the absence of any commercial or financial relationships that could be construed as a potential conflict of interest.

## Publisher’s Note

All claims expressed in this article are solely those of the authors and do not necessarily represent those of their affiliated organizations, or those of the publisher, the editors and the reviewers. Any product that may be evaluated in this article, or claim that may be made by its manufacturer, is not guaranteed or endorsed by the publisher.
