# Supplementary material for: Associations of Sperm mtDNA Copy Number, DNA Fragmentation Index, and Reactive Oxygen Species With Clinical Outcomes in ART Treatments
Source: Front Endocrinol (Lausanne). 2022 Mar 23;13:849534. doi: 10.3389/fendo.2022.849534 (PMC8983846; doi:10.3389/fendo.2022.849534)
Supplement: Supplementary file 1 [file DataSheet_1.docx]

Supplementary Material

**Associations of** **sperm mtDNA copy number, DNA fragmentation index, and reactive oxygen species with clinical outcomes in ART treatments**

**Supplementary Figure 1. Correlations between male age, mtDNA-CN, ROS/MS, DFI and sperm parameters.**


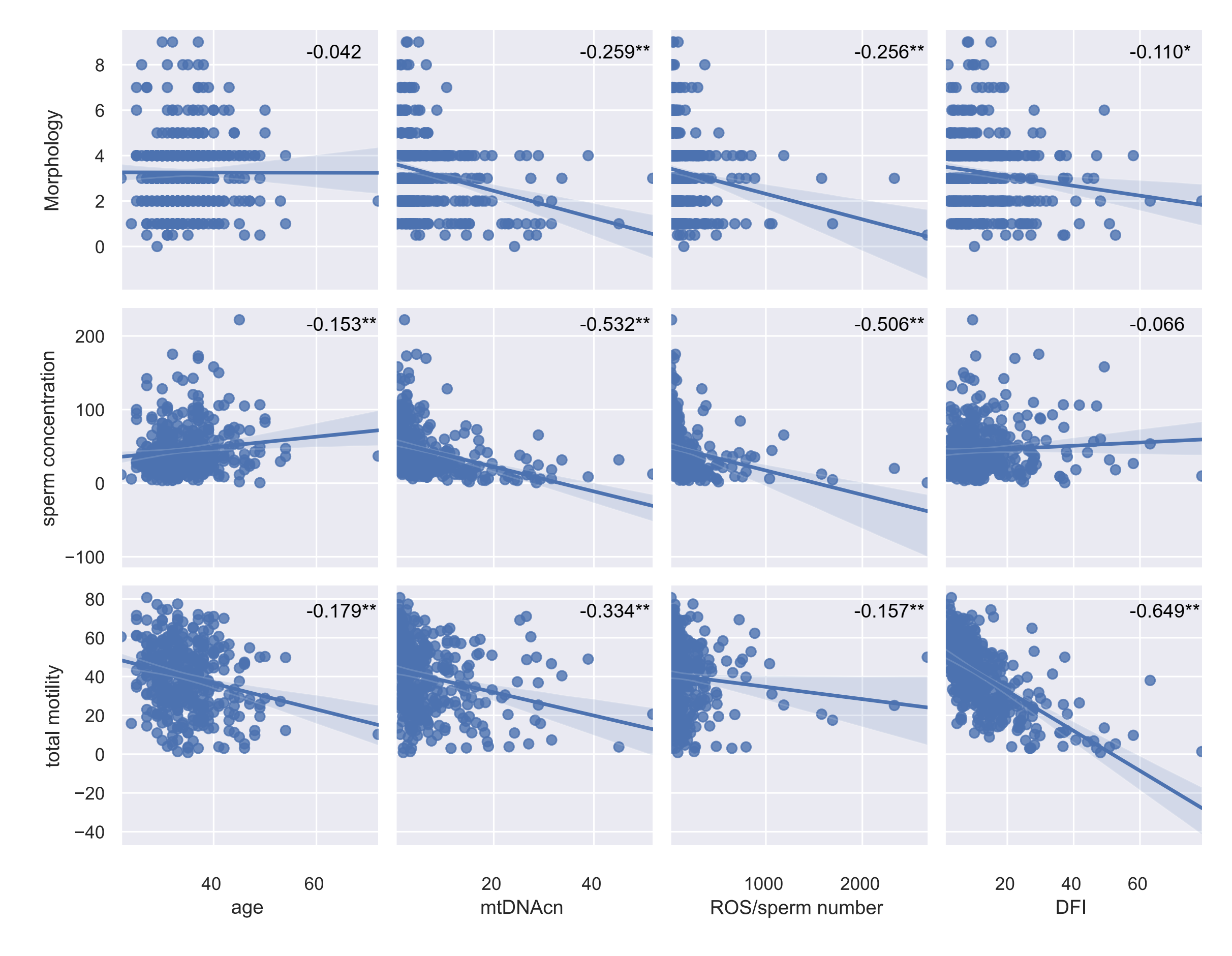


mtDNA-CN: mitochondrial DNA copy number; ROS: reactive oxygen species; DFI: DNA fragmentation index; *: *P* < 0.05; **: *P* < 0.01

**Supplementary Table 1. Mitochondrial PCR primer/probe sequences.**

|  | Binding site positions | Sequence |
| --- | --- | --- |
| Minor arc |  |  |
| Forward | mt 16 528–16 548 | 5′- CTA AAT AGC CCA CAC GTT CCC-3′ |
| Reverse | mt 23–42 | 5′- AGA GCT CCC GTG AGT GGT TA-3′ |
| Probe | mt 16 560–10 | 6FAM-CAT CAC GAT GGA TCA CAG GT-MGBNFQ |

**Supplemental Table 2. Protocols of controlled ovarian stimulation in ART cases.**

|  | GnRH-A (n=80) | GnRH-a (n=19) | Mild stimulation (n=27) | *P* |
| --- | --- | --- | --- | --- |
| Female age | 32 (29, 34) | 33 (30, 37) | 38 (35, 43) | <0.001 |
| Male age | 32 (30, 36) | 36 (31, 38) | 37 (34, 43) | <0.001 |
| Percentage of male with abnormal sperms (%) | 45% | 68% | 81% | 0.002 |
| Morphology, % | 3 (2, 4) | 3 (1, 4) | 3 (1, 3) | 0.223 |
| Sperm concentration, million/ml | 38.87 (22.96, 53.69) | 38.32 (13.67, 87.30) | 32.40 (24.07, 52.97) | 0.753 |
| Total motility, % | 42.95 (27.83, 52.20) | 38.4 (25.90, 50.40) | 32.90 (19.40, 49.60) | 0.134 |
| mtDNA-CN | 3.62 (2.19, 6.85) | 3.45 (1.96, 6.20) | 3.45 (2.26, 12.29) | 0.853 |
| ROS/MS | 78.28 (49.34, 168.13) | 56.48 (34.92, 93.53) | 78.69 (50.34, 140.08) | 0.281 |
| DFI | 8.76 (5.36, 15.19) | 11.84 (6.21, 18.84) | 8.83 (5.36, 18.39) | 0.488 |
| Fertilization rate (%) | 74.64 (50.00, 86.20) | 66.67 (50.00, 81.82) | 85.71 (66.67, 100.00) | 0.035 |
| Cleavage rate (%) | 100.00 (100.00, 100.00) | 100.00 (95.24, 100.00) | 100.00 (100.00, 100.00) | 0.599 |
| Top-quality embryo rate (%) | 42.86 (21.25, 66.67) | 35.00 (0.00, 50.00) | 33.33 (0.00, 50.00) | 0.115 |

The data were analyzed with Kruskal-Wallis test. GnRH-A, gonadotrophin-releasing hormone antagonist protocol; GnRH-a, gonadotrophin-releasing hormone agonist protocols (long, short, and ultra-long protocols).

**Supplemental Table 3.** **Associations of mtDNA-CN, DFI, and ROS with seminal quality.**

|  | Seminal quality | |
| --- | --- | --- |
|  | OR (95% CI) | *P* |
| mtDNA-CN | 1.124 (1.062, 1.189) | 5.1x10^-5^ |
| DFI | 1.129 (1.082, 1.177) | 1.9x10^-8^ |
| ROS/MS | 1.002 (1.000, 1.003) | 0.034 |

mtDNA-CN: mitochondrial DNA copy number; ROS/MS: reactive oxygen species per million sperms; DFI: DNA fragmentation index; OR: odds ratio; CI: confidence interval;

**Supplemental Table 4. Embryotic outcomes of patients with the first IVF/ICSI cycle.**

|  | Fertilization　rate (%) | | Cleavage rate (%) | | Top-quality embryo rate (%) | |
| --- | --- | --- | --- | --- | --- | --- |
|  | Coefficients (95% CI) | *P* | Coefficients (95% CI) | *P* | Coefficients (95% CI) | *P* |
| mtDNA-CN | -0.827 (-1.450, -0.205) | 0.010 | 0.225 (-0.312, 0.761) | 0.408 | 0.230 (-0.526, 0.986) | 0.547 |
| DFI | -0.674 (-1.260, -0.087) | 0.025 | 0.084 (-0.586, 0.419) | 0.741 | 0.412 (-0.290, 1.113) | 0.247 |
| ROS/MS | -0.011 (-0.039, 0.017) | 0.448 | 0.006 (-0.017, 0.030) | 0.523 | 5.785x10^-6^(-0.033, 0.033) | 1.000 |

The data are analyzed with linear regression (n=92). ART: assisted reproductive technology; mtDNA-CN: mitochondrial DNA copy number; ROS/MS: reactive oxygen species per million sperms; DFI: DNA fragmentation index; CI: confidence interval;

**Supplemental Table 5. Analysis of ART outcomes in patients with the first IVF/ICSI cycle.**

|  |  | Fertilization　rate | | | |
| --- | --- | --- | --- | --- | --- |
|  |  | OR | 95% CI | | *P* |
| mtDNA-CN | Q1 | reference | | | |
|  | Q2 | 0.945 | 0.833 | 1.073 | 0.386 |
|  | Q3 | 0.984 | 0.868 | 1.115 | 0.803 |
|  | Q4 | 0.881 | 0.762 | 1.019 | 0.087 |
| DFI |  | 0.995 | 0.989 | 1.002 | 0.143 |
| ROS/MS |  | 1.000 | 1.000 | 1.000 | 0.655 |
| Male age |  | 0.998 | 0.984 | 1.012 | 0.770 |
| Female age |  | 1.012 | 0.996 | 1.027 | 0.141 |
| Sperm parameters | normal | reference |  |  |  |
|  | abnormal | 0.915 | 0.826 | 1.013 | 0.087 |
| ART strategy | IVF | reference |  |  |  |
|  | ICSI | 0.923 | 0.831 | 1.024 | 0.130 |
| COS protocols | GnRH-a | reference |  |  |  |
|  | GnRH-A | 1.019 | 0.894 | 1.162 | 0.774 |
|  | MS | 1.025 | 0.876 | 1.198 | 0.760 |
| Retrieved oocytes | Q1 (0-3) | reference |  |  |  |
|  | Q2 (4-7) | 0.959 | 0.828 | 1.112 | 0.582 |
|  | Q3 (8-12) | 1.007 | 0.861 | 1.178 | 0.929 |
|  | Q4 (13-44) | 0.992 | 0.852 | 1.153 | 0.912 |

The data are analyzed with generalized linear model (n=92). IVF: in vitro fertilization; ICSI: intracytoplasmic sperm injection; mtDNA-CN: mitochondrial DNA copy number; ROS/MS: reactive oxygen species per million sperms; DFI: DNA fragmentation index; COS, controlled ovarian stimulation; GnRH-a, gonadotrophin-releasing hormone agonist protocols, including long, short, and ultra-long protocols; GnRH-A, the GnRH antagonist protocol; MS, mild ovarian stimulation. OR: odds ratio; CI: confidence interval; **P* < 0.05.

**Supplemental Table 6. Summary of ART comes.**

|  | IVF | ICSI | *P* value |
| --- | --- | --- | --- |
| Female median age (IQR) | 32 [28, 34] | 33 [30.25, 34.75] | 0.109 |
| Male median age (IQR) | 32 [30, 36] | 34.5 [31.25, 38] | 0.217 |
| NO. of retrieved oocytes | 828 | 377 |  |
| NO. of fertilized embryos | 583/828 | 241/377 | 0.025* |
| NO. of cleavage-stage embryos | 560/583 | 231/241 | 0.892 |
| NO. of good quality embryos | 234/583 | 108/241 | 0.215 |
| NO. of patients with transfer | 59 | 20 |  |
| NO. of transfers | 90 | 24 |  |
| Fresh (%) | 11/90 | 1/20 | 0.455 |
| Frozen (%) | 79/90 | 23/24 |  |
| NO. of clinical pregnancy/transfer | 32/90 | 8/24 | 0.839 |
| NO. of Ongoing pregnancy/transfer | 28/90 | 7/24 | 0.854 |
| NO. of Live births/transfer | 25/90 | 6/24 | 0.786 |
| NO. of miscarriages | 3 | 1 |  |
| NO. of ectopic pregnancy | 1 | 0 |  |

IVF: in vitro fertilization; ICSI: intracytoplasmic sperm injection; IQR: inter-quartile range, * P < 0.05.

**Supplemental Table 7. Analysis of pregnancy outcomes with generalized estimating equations.**

|  | |  |  | Clinical pregnancy | | Live birth | | |
| --- | --- | --- | --- | --- | --- | --- | --- | --- |
|  | |  |  | Adj OR (95% CI) | *P* | | Adj OR (95% CI) | *P* |
| mtDNA-CN |  | | | 0.971 (0.903, 1.043) | 0.419 | | 1.003 (0.937, 1.074) | 0.936 |
| DFI |  | | | 1.025(0.971, 1.081) | 0.375 | | 1.020 (0.961, 1.082) | 0.514 |
| ROS/MS |  | | | 1.002 (0.999, 1.005) | 0.121 | | 1.000 (0.997, 1.003) | 0.956 |
| Embryo transfer | ET | | | reference |  | |  |  |
|  | FET | | | 1.070 (0.301, 3.804) | 0.917 | | 1.445 (0.335, 6.230) | 0.622 |
| Retrieved oocytes | Q1 (0-6) | | | reference |  | |  |  |
|  | Q2 (7-10) | | | 0.708 (0.204, 2.457) | 0.587 | | 1.019 (0.266, 3.901) | 0.978 |
|  | Q3 (11-15) | | | 0.481 (0.123, 1.891) | 0.295 | | 0.654 (0.149, 2.875) | 0.574 |
|  | Q4 (15-44) | | | 1.467 (0.396, 5.438) | 0.566 | | 1.257 (0.308, 5.133) | 0.750 |
| ART strategy | IVF | | | reference |  | |  |  |
|  | ICSI | | | 0.808 (0.259, 2.527) | 0.715 | | 0.724 (0.204, 2.571) | 0.618 |
| COS protocols | GnRH-a | | | reference |  | |  |  |
|  | GnRH-A | | | 1.263 (0.451, 3.535) | 0.657 | | 0.995 (0.306, 3.238) | 0.993 |
|  | MS | | | 0.133 (0.009, 1.974) | 0.143 | | 0.222 (0.014, 3.447) | 0.282 |
| IVF/ICSI cycle rank | Rank 1 | | | reference |  | |  |  |
|  | Rank >1 | | | 0.925 (0.221, 3.882) | 0.915 | | 1.086 (0.217, 5.427) | 0.920 |
| Sperm parameters | normal  abnormal | | | reference |  | |  |  |
|  | abnormal | | | 1.472 (0.603, 3.588) | 0.396 | | 1.667 (0.618, 4.498) | 0.313 |
| Male age |  | | | 0.931 (0.777, 1.114) | 0.434 | | 0.977 (0.804, 1.186) | 0.812 |
| Female age |  | | | 1.067 (0.920, 1.237) | 0.394 | | 0.994 (0.852, 1.161) | 0.941 |

Adj OR is adjusted for male age, female age, seminal quality, frozen/fresh embryo transfer, ART strategy, IVF cycle rank, COS protocols, number of retrieved oocytes; OR: odds ratio; CI: confidence interval.
